# Case-Fatality Ratio of Blood Culture–Confirmed Typhoid Fever in Dhaka, Bangladesh

**DOI:** 10.1093/infdis/jiy543

**Published:** 2018-10-09

**Authors:** Alexander T Yu, Nuhu Amin, Muhammad Waliur Rahman, Emily S Gurley, Kazi Mizanur Rahman, Stephen P Luby

**Affiliations:** 1 Stanford University, California; 2 International Centre for Diarrhoeal Disease Research, Bangladesh, Dhaka; 3 Johns Hopkins Bloomberg School of Public Health, Baltimore, Maryland; 4 Public Health and Tropical Medicine, College of Public Health, Medical and Veterinary Sciences, James Cook University, Townsville, Queensland, Australia

**Keywords:** typhoid, enteric fever, case-fatality ratio, global burden of disease, laboratory-based surveillance

## Abstract

With impending rollout of new conjugate typhoid vaccines, better estimates of typhoid case-fatality ratio are needed for countries to set priorities for public health programs. We enrolled 1425 patients of all ages with blood culture–confirmed *Salmonella* Typhi from laboratory networks serving inpatients and outpatients in Dhaka, Bangladesh. Participants were asked about symptoms and complications including death experienced over a median 3-month period following blood culture diagnosis. Four fatal cases were identified (case-fatality ratio of 0.3% [95% confidence interval, .05%–.55%]). Applying this case-fatality ratio to global typhoid burden estimates would reduce deaths by 70%.

Typhoid fever is a systemic infection caused by *Salmonella enterica* serovar Typhi that can lead to severe outcomes including death [1]. The case-fatality ratio (the proportion of people infected with *S.* Typhi who die as a result of infection) remains poorly characterized [2]. Few studies address case-fatality ratio, and they provide wide-ranging estimates that vary depending on study methodology [2–4]. An accurate case-fatality ratio is important as it contributes proportionally to disease burden estimates and thus to cost-effectiveness analyses, making it crucial for policy, public health, and resource allocation decisions.

In 2004 and 2008, Crump et al reviewed available literature to estimate the global burden of typhoid and noted a dearth of data to estimate case-fatality ratio. Available data had wide ranging results depending on if they were derived from community or hospital-based studies [5]. Community-based studies had a median case-fatality ratio of 0% (range, 0–1.8%), while hospital-based studies that included patients of all ages had a higher median case-fatality ratio of 3.1% (range, 0–13.9%) [6]. Similarly, a 2015 review found an overall hospital-based case-fatality ratio of 2% compared to 0% in community-based studies [7]. Based on available data and the opinion that typhoid cases mainly occur in the community rather than in the hospital, Crump et al settled on a 1% case-fatality ratio that has since been widely cited [5].

Differences in results from community- vs hospital-based studies highlight the difficulty in establishing an accurate case-fatality ratio when focusing on either population alone. Each captures a subset of patients and they likely represent upper and lower limits rather than a representative ratio. To capture the experiences of both populations, we estimated case-fatality ratio from laboratory networks utilized by outpatients and by hospitalized inpatients.

## METHODS

Between January and December 2010, we recruited patients with *S*. Typhi diagnosed by blood culture, identified from 6 large, private laboratory networks serving Dhaka, Bangladesh. Laboratory networks were selected based on high volume (200–300 blood cultures per month), presence of a supervising microbiologist, and service to hospitals as well as outpatient pharmacies and clinics. Laboratories performed blood cultures on patients irrespective of formal referral from a doctor.

Each laboratory kept a log of all patients obtaining blood cultures during the study period. Study personnel visited laboratories weekly, obtaining contact information and antibiotic resistance testing results for patients with blood cultures positive for *S*. Typhi who gave permission to be contacted. Study personnel then called eligible participants to explain the study, obtain informed consent, and administer a questionnaire. Follow-up information was obtained through a second and third phone interview, attempted at 2-week intervals after the initial questionnaire. For enrollment and follow-up, study personnel tried a minimum of 9 times to reach patients or their relatives.

Inclusion criteria were a blood culture–positive for *S.* Typhi at a study laboratory and residence within Dhaka city, with no age restrictions. The head of household was interviewed for adults too sick to respond and for children <18 years of age.

Data from the Bangladesh Bureau of Statistics were used for demographic comparisons [8]. Two laboratory networks used international guidelines for antimicrobial susceptibility testing (Clinical Laboratory and Standards Institute and European Committee on Antimicrobial Susceptibility Testing); the other 4 used internal guidelines.

The study protocol was approved by the International Centre for Diarrhoeal Disease Research, Bangladesh (icddr,b) ethical review committee.

### Statistical Analyses

Assuming a 1% typhoid fever case-fatality ratio [5], a 0.5% margin of error, and a 95% confidence interval (CI), we aimed to enroll 1521 patients with typhoid.

All enrolled participants were included in the final analyses. Simple descriptive statistics were performed to compare frequencies and proportions. Bivariate analyses were done using the χ^2^ test with statistical significance set at *P* < .05. Sensitivity analysis was done to assess the impact that deaths among participants lost to follow-up would have on case-fatality ratio.

## RESULTS

A total of 1425 patients were enrolled, all of whom answered an initial interview a median of 6.6 (interquartile range [IQR], 4.7–8.3) weeks after blood cultures were drawn. Data on the number of eligible participants not enrolled, either because they were unable to be contacted or declined participation, were not available. At the second interview (median, 8.9 [IQR, 7.1–10.7] weeks after culture), 44 (3.1%) participants were lost to follow-up; at the third and final interview (median, 11.4 [IQR, 9.4–13.4] weeks after culture), an additional 32 (2.2%) were lost. Participants were mostly male (59%) and had a median age of 14 years (range, 6 months–64 years). Compared to the general urban Dhaka population, the study cohort had more resources (5% vs 57% owning a computer, 40% vs 86% owning a refrigerator), had fathers with higher levels of education (26% vs 72% with high school or higher level of schooling), and had more access to piped water (58% vs 90%) and a flush toilet (59% vs 74%) [8] (Table 1). Patients lost to follow-up were less likely to drink boiled water (81% vs 90%; *P* = .01) and more likely to use a toilet with a septic tank (33% vs 19%; *P* = .01).

**Table 1.** Demographics of Enrolled Patient Population and Symptoms Experienced by Hospitalized and Nonhospitalized Patients

Characteristic		All (N = 1425)	Hospitalized (n = 370 [26%])	Not Hospitalized (n = 1051 [74%])	*P* Value	Died (n = 4 [0.3%])
Demographic characteristics						
Sex						
Male		839 (59)	212 (57)	627 (60)	.43	0 (0)
Female		586 (41)	158 (43)	424 (40)		4 (100)
Age, y						
Age, y, median (IQR)		14 (5–23)	13 (2–21)	14 (6–23)	.91	Ages: 32, 39, 43, 65
No. <5 y old (% of population)		304 (21)	97 (26)	207 (20)	.37	0 (0)
Range		0.6–64	0.75–60	0.6–64		
Household characteristics						
Education level of father					.46	
	No school	47 (3.3)	15 (4)	31 (3)		1 (25)
	Less than high school	329 (23)	77 (21)	250 (24)		2 (50)
	High school graduate	196 (14)	49 (13)	147 (14)		0 (0)
	College or above	828 (58)	220 (60)	607 (58)		1 (25)
	Other	25 (2)	9 (2)	16 (2)		
Own a motor car		245 (17)	67 (18)	178 (17)	.81	1 (25)
Computer/laptop in house		811 (57)	209 (56)	602 (57)	.95	1 (25)
Refrigerator in house		1219 (86)	319 (86)	900 (86)	.82	4 (100)
Water source						
	City water supply	1284 (90)	334 (90)	950 (90)	.88	3 (75)
	Tube well	14 (1)	5 (1)	9 (1)	.62	1 (25)
	Drink boiled water	1266 (89)	328 (89)	938 (89)	.86	2 (50)
Use gas for cooking		1255 (88)	336 (91)	919 (87)	.17	3 (75)
Own home or land in Dhaka		589 (41)	164 (44)	425 (40)	.09	1 (25)
Main toilet is a flush toilet		1061 (74)	291 (79)	770 (73)	.18	2 (50)
Symptoms experienced by patient						
Fever		1389 (97)	368 (99)	1017 (97)	.004	
Vomiting		601 (44)	188 (53)	413 (40)	<.001	
Blood in stool		31 (2)	10 (3)	21 (2)	.59	
Abdominal pain		291 (21)	102 (28)	189 (18)	<.001	
Abdominal distension		57 (4)	28 (8)	29 (3)	<.001	
Fainted		46 (3)	20 (6)	26 (3)	.02	
Had convulsion or seizure		45 (3)	22 (6)	23 (2)	.001	
Hypothermia of hand or foot		181 (13)	70 (20)	111 (11)	<.001	
Psychological stress		38 (3)	20 (6)	18 (2)	<.001	

Data are presented as No. (%) unless otherwise indicated.

Abbreviation: IQR, interquartile range.

We identified 4 fatal cases (0.3% [95% CI, .05%–.55%). All were adult females and were hospitalized for typhoid fever; 3 died in the hospital, 1 died immediately after leaving the hospital, and all died within 3 days of blood culture collection. Among hospitalized patients, the case-fatality ratio was 1.1% (95% CI, 0.02%–2.1%) and 0% in outpatients. Compared to survivors, patients who died had fewer resources and had fathers with less education (Table 1). Sensitivity analysis for a scenario assuming that all participants lost to follow-up did so because of death increases the case-fatality ratio to 5.6%.

Among all participants, the most common symptoms experienced were fever, vomiting, and abdominal pain. Potentially more severe symptoms, including blood in stool or seizures, were reported in <5%. No intestinal perforations were reported (Table 1).

### 

#### Antibiotic Resistance

Testing for all 3 historical first-line drugs for typhoid (ampicillin, chloramphenicol, and cotrimoxazole) was done in 661 (53%) isolates. Of those tested, 175 (26%) were resistant to all 3. Azithromycin resistance was reported in 38% of isolates tested (Tables 2 and 3).

**Table 2.** Antibiotic Usage Among Patients

Antibiotic Usage	All (N = 1425)	Hospitalized (n = 370)	Not Hospitalized (n = 1051)	*P* Value	Died (n = 4)
Started antibiotics					
Before blood culture	665 (47)	199 (54)	463 (44)	.05	3 (75)
After blood culture but before results reported	238 (17)	70 (19)	167 (16)	.45	1 (25)
After blood culture and resistance report	515 (36)	100 (27)	415 (40)	<.001	0
Unknown	2 (0.1)	0	2 (0.2)	…	0

Data are presented as No. (%).

**Table 3.** Antimicrobial Susceptibility Testing of *Salmonella* Typhi Blood Culture Isolates Performed at 6 Dhaka, Bangladesh–Based Laboratories

Antibiotic	Antibiotic Resistance, No. (% of Tested Isolates)			
	No. of Isolates Tested	Sensitive	Intermediate	Resistant
Ampicillin	872	499 (57)	3 (0)	370 (42)
Azithromycin	1157	374 (32)	348 (30)	435 (38)
Cefixime	1250	1168 (93)	71 (6)	11 (1)
Ceftriaxone	1254	1253 (>99)	…	1 (0)
Chloramphenicol	870	677 (78)	…	193 (22)
Ciprofloxacin	1239	966 (73)	175 (14)	98 (8)
Cotrimoxazole	1238	902 (73)	…	336 (27)
Nalidixic acid	1193	25 (2)	2 (0)	1166 (98)

## DISCUSSION

We found an overall case-fatality ratio of 0.3% (95% CI, .05%–.55%) for patients with blood culture–confirmed *Salmonella* Typhi, identified through 6 laboratory networks serving inpatients and outpatients in Dhaka, Bangladesh. This is considerably lower than the 1% estimated by Crump et al. While the 1% estimate tried to balance findings from studies focused on either community or hospital patients alone, our study attempted to directly capture both populations at the same time. Using a broader surveillance method, this study adds an important data point on typhoid case-fatality ratio for an endemic region.

Community-based studies often take an active, population-based approach, where a community is repeatedly surveyed for typhoid. These studies directly measure incidence by trying to capture all cases, including mild ones that otherwise would not present to the healthcare system. The intensity and expense of these studies make them uncommon and geographically limited [7, 9]. They also interrupt the natural history of disease because earlier diagnosis leads to earlier treatment, preventing more severe sequelae and artificially lowering case-fatality ratios [9, 10].

In contrast, hospital-based estimates are logistically simpler but biased toward patients with more severe symptoms requiring hospital-level care and those with the resources to access such care [9, 10]. They risk missing milder cases that stay in the community and patients who cannot access care or otherwise seek care in the informal sector [9, 11]. By underestimating the total number of cases in the population, they result in artificially high case-fatality estimates.

The limitations of each approach should be taken into consideration when designing research studies or planning public health policy. Community-based studies underestimate case fatalities and thus would provide limited evidence on lives saved through vaccination. Hospital-based estimates of case fatalities or disease severity, by contrast, are difficult to extrapolate to the broader community.

By utilizing a laboratory network serving both the community and hospitals and thus expanding the population represented without altering the natural course of disease, our study attempts to improve estimates for both the numerator (total number of fatal cases) and the denominator (total number of typhoid cases) used to calculate case-fatality ratio. Applying our ratio of 0.3% (95% CI, .05%–.55%) to the 2012 estimate of 26.9 million global cases of typhoid suggests 80700 (95% CI, 13450–147950) annual deaths, much lower than the 269000 deaths estimated using the 1% case-fatality ratio by Crump et al, or the half a million deaths using the 2% case-fatality ratio found by Azmatullah et al [3, 5, 7]. These numbers have important implications for national and global decision making, from the allocation of limited public health dollars to vaccine implementation and healthcare infrastructure development.

Children in our study bore a disproportionate burden of typhoid disease. Children <5 and <15 years old accounted for 21% and 51% of all cases, even though they account for 10% and 30% of the urban Bangladeshi population, respectively [8]. This is similar to age-trends reported in the literature, though less than what was found in a study of Dhaka slums where 60% of typhoid cases were in children <5 years old [12]. Despite this higher case burden, we found no fatal cases among children, consistent with systematic reviews that have found lower case-fatality ratios in children compared to adults [6, 7].

Our data also suggest that those who die from typhoid are poorer than those who survive, and that those able to obtain cultures have more resources than the general population. Socioeconomic status has been found to be the most important determinant in healthcare-seeking behavior in Bangladesh [13]. Patients lacking resources are the least likely to seek care, have blood cultures collected, or be treated in hospitals, and are the most likely to suffer severe outcomes including death. By incompletely capturing this population, we are both underrepresenting the total number of typhoid cases as well as the total number of fatal cases in the population. The exact ratio of these missed cases is unclear and could lower or raise the true population case-fatality ratio.

An important limitation in our study is that fatal cases of typhoid are at risk of not having been enrolled in the first place. To address this limitation, we made multiple attempts to contact family members of eligible participants if they could not be directly reached. All 4 fatal cases in our study died before initial contact but were able to be identified this way. Despite this, it is possible that fatal cases were missed at enrollment. Data on how many eligible patients were not enrolled was not consistently maintained at the different laboratories, preventing sensitivity analysis.

A participant who dies during the study would similarly be at risk of being lost to follow-up, regardless of attempts at family contact. While most (95%) completed a median 3 months of follow-up, 76 people were lost before the end of the study. However, since initial enrollment was a median of 6.6 weeks after blood culture, we had more than a month of data for the majority enrolled. This time period includes the acute typhoid fever period when the majority of deaths in the cohort would have been expected to occur [14]. Consistent with this was that all 4 fatal cases in our study died prior to enrollment, only 3 days from time of blood culture. Still, even a small number of missed deaths would have a large impact on the calculated case-fatality ratio, and sensitivity analysis show that case-fatality ratio could range from 0.3% to 5.6%.

While we found high azithromycin resistance, the heterogeneity of laboratory techniques used limit our ability to interpret this data.

Case-fatality ratio likely varies regionally and is influenced by healthcare access, resources and infrastructure, antimicrobial resistance patterns, and socioeconomics. Studies to assess case fatality in other countries and settings could help inform regional and global typhoid burden estimates [6, 9]. Attention should be focused on outpatients (where typhoid disease is concentrated) and on people with lower socioeconomic status or that seek care in the informal sector (where patients are at higher risk for poor outcomes) [12, 13]. Our 0.3% case-fatality ratio utilizes a laboratory-based network serving both outpatients and inpatients but misses those not accessing the formal health sector. It provides an additional point estimate of this important parameter, and offers a cost-effective method to replicate. With the potential for both vaccines and antimicrobial resistance to alter typhoid morbidity and mortality, an accurate and continually updated understanding of case-fatality ratio is important to update global burden estimates and for national and global policy making [15].

## Notes

Presented in part: American Society of Tropical Medicine and Hygiene Conference, Baltimore, Maryland, 2017; and 10th International Conference on Typhoid and Other Salmonelloses, Kampala, Uganda, 2017.

## Notes

***Acknowledgments.*** #8195;The authors gratefully acknowledge the data collectors involved with this study and the authorities at the diagnostic centers for their collaboration. The authors also gratefully acknowledge John Crump and Eric Mintz for their thoughtful review during protocol development. icddr,b acknowledges with gratitude the commitment of the Centers for Disease Control and Prevention (CDC) to its research efforts, as well as the governments of Bangladesh, Canada, Sweden, and the United Kingdom for providing core/unrestricted support to icddr,b.

***Financial support.*** This research protocol was funded by the CDC (grant number 081126).

***Supplement sponsorship.*** This article is part of the supplement “Surveillance for Enteric Fever in Asia Project (SEAP),” sponsored by the Sabin Vaccine Institute.

***Potential conflicts of interest.*** All authors: No reported conflicts of interest. All authors have submitted the ICMJE Form for Disclosure of Potential Conflicts of Interest. Conflicts that the editors consider relevant to the content of the manuscript have been disclosed.
